# Malaria in Southeastern China from 2012 to 2016: Analysis of Imported Cases

**DOI:** 10.4269/ajtmh.17-0476

**Published:** 2018-02-26

**Authors:** Xuan Zhang, Linong Yao, Jimin Sun, Jinren Pan, Hualiang Chen, Lingling Zhang, Wei Ruan

**Affiliations:** Zhejiang Provincial Center for Disease Control and Prevention, Hangzhou, PR China

## Abstract

To study the epidemiological distribution and the incident trends of imported malaria from 2012 to 2016 in Zhejiang Province, southeastern China, we collected data on malaria from the Information System for Parasitic Disease Control and Prevention. A total of 1,003 malaria cases were reported during 2012–2016, and all of these cases were imported. *Plasmodium falciparum* was the predominant species (76.3%) in Zhejiang Province. The percentage of *Plasmodium vivax* decreased from 33.6% to 8.1%, whereas the percentage of *Plasmodium ovale* and *Plasmodium malariae* increased. Most cases were male (89.8%), mostly in the age group of 21–50 years (82.6%). Businessmen (33.0%), workers (21.0%), farmers (18.8%), and overseas laborers (11.7%) were at high risk. The origin of the largest number of imported cases was Africa (89.5%), followed by Asia (10.0%) and Oceania (0.5%). The time interval from illness onset to confirmation was found to be significantly associated with the complications of patients. Out of 3,461 febrile individuals tested during reactive case detection, 10 malaria-positive individuals were identified. Effective surveillance and response system should be strengthened to prevent the reintroduction of malaria.

## INTRODUCTION

Malaria is a life-threatening disease caused by parasites that are transmitted to people through the bites of infected female *Anopheles* mosquitoes. It remains one of the major public health problems in the world. According to the estimation by the World Health Organization, 91 countries and territories had ongoing malaria transmission in 2015, and about 32 billion people (nearly half of the world’s population) were at risk of malaria. There were 212 million cases of malaria and 429,000 deaths worldwide in 2015.^1^ China has made progress in controlling locally transmitted malaria over the past decades, which led to a dramatic decrease in the incidence of malaria. The total malaria incidence rate in China had reduced to 1.06/100,000 in 2009.^2^ Since 2010, China has initiated the National Malaria Elimination Program, aiming to eliminate indigenous malaria except for border areas by 2015 and achieve malaria elimination nationwide by 2020.^3^

With the conduct of comprehensive measures, including treatment and management of infection source, control and prevention of mosquitoes, and indoor residual spraying, the last autochthonous cases of malaria in Zhejiang Province occurred in 2011.^4^ However, in recent years, imported malaria has been an increasing problem in Zhejiang Province, and little is known about the changing characteristics of these malaria cases. To understand and address this challenge, this study analyzed the epidemiological distribution and trends of imported malaria in Zhejiang Province from 2012 to 2016.

## MATERIALS AND METHODS

### Case definition.

Laboratory-confirmed cases were diagnosed by microscopy, rapid diagnostic tests (RDTs), and polymerase chain reaction (PCR).^5^ Patients with malaria-like symptoms and epidemiological history but no detectable parasites in blood samples were not included in the analysis. According to the Technical Scheme of Malaria Elimination in China,^5^ imported cases were defined as patients with malaria infections traced to origins in a malaria-endemic country within the previous month or those whose had traveled to another district in China with clear evidence of malaria transmission (local cases reported). Indigenous cases were defined as patients infected in the province with no history of travel and unfalsifiable locally acquired transmission.

### Data collection.

Malaria is a class “B” notifiable disease in China, and physicians in hospitals are required to report cases to the China Information System for Diseases Control and Prevention (CISDCP) within 24 hours of diagnosis. The China Information System for Diseases Control and Prevention only contains basic information of malaria cases such as demographic information, illness onset date, and reporting institutions. On the basis of CISDCP, malaria system of the Information System for Parasitic Disease Control and Prevention (ISPDCP) was expanded by the National Institute of Parasitic Diseases to collect clinical information (symptoms onset date, diagnosis date, and treatment), epidemiological investigation, and reactive case detection (RACD) information from the patients. All laboratory-confirmed cases were investigated in person and information was reported to ISPDCP.

### Statistical analysis.

Data were analyzed using the Statistical Package for the Social Sciences (SPSS v16.0; SPSS Inc., Chicago, IL). χ^2^ test was used to compare count data. Nonparametric test (Kruskal–Wallis test) was used for non-normally distributed or heterogeneous data. A multiple linear model was used to modify the potential factor effects. A *P* value of less than 0.05 was considered statistically significant.

## RESULTS

**Figure 1.**
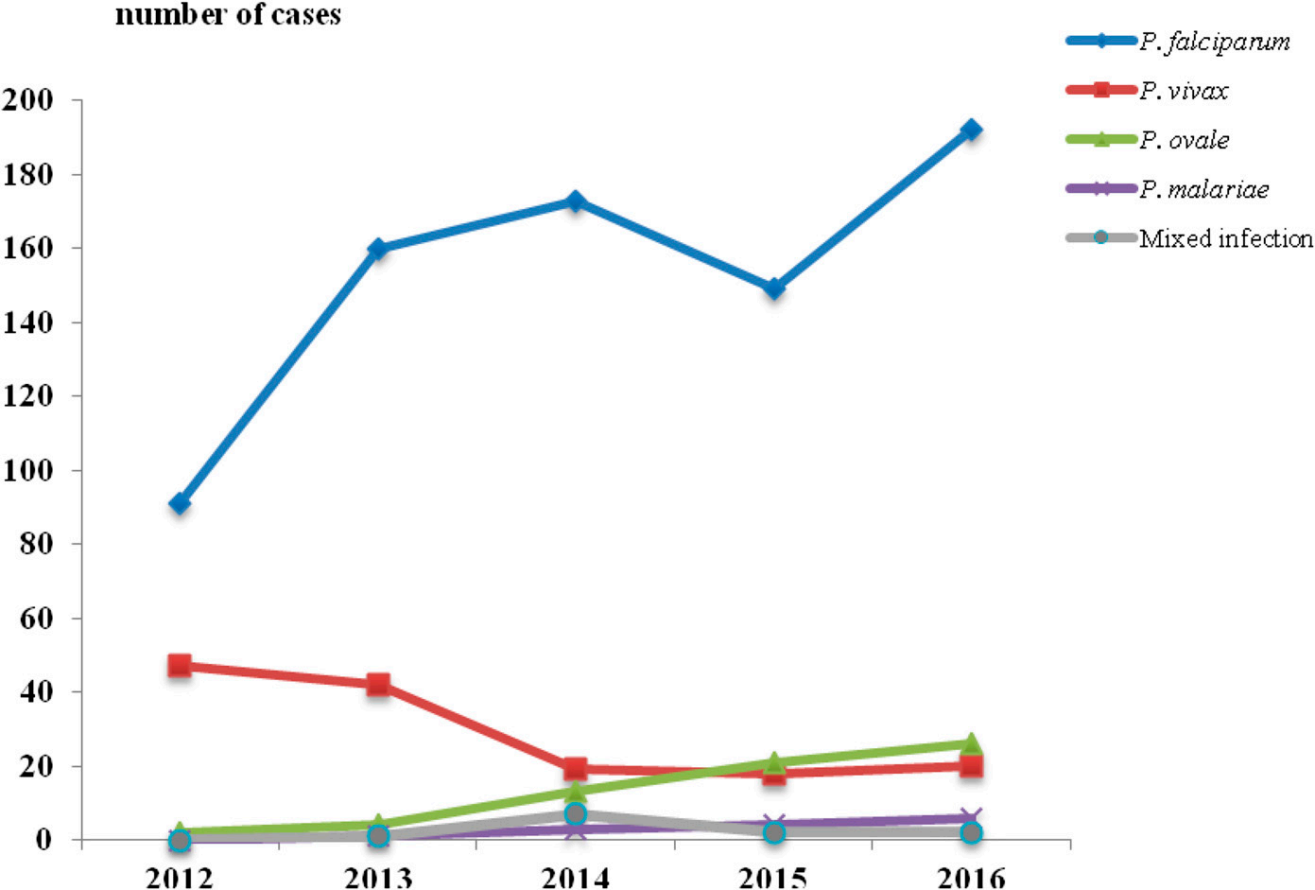
Annual distribution of total malaria cases in Zhejiang Province, 2012–2016.

During the period of 2012–2016, a total of 1,003 malaria cases were identified and reported in Zhejiang Province (Figure 1). All of these cases were imported from other provinces of China or other countries. *Plasmodium falciparum* was the predominant species, which accounted for 76.3% (765/1,003) of the total malaria cases, and the percentage peaked in 2014 to 80.5%. Of note, two patients died of *P. falciparum* in 2013 and 2016. The percentage of *Plasmodium vivax* decreased significantly from 33.6% in 2012 to 8.1% in 2016 (χ^2^ = 40.259, *P* < 0.001), whereas the percentage of *Plasmodium ovale* increased significantly from 1.4% in 2012 to 10.6% in 2016 (χ^2^ = 11.080, *P* < 0.001) and the percentage of *Plasmodium malariae* increased from 0.5% in 2013 to 2.4% in 2016 (χ^2^ = 2.847, *P* > 0.05). Moreover, six imported cases were due to relapsing *P. vivax* and *P. ovale* that had been acquired several months up to about 2 years before. Mixed infections with two species were also reported in the past 5 years, which accounted for 1.2% of all malaria cases.

Of all the malaria cases, 89.8% (901/1,003) cases were male, and the male-to-female ratio was 8.8:1 (901:102). Occupations of patients ranged from businessmen (331 cases, 33.0%), workers (193 cases, 19.2%), farmers (207 cases, 20.6%), overseas laborers (117 cases, 11.7%), others (96 cases, 9.6%), houseworkers (34 cases, 3.4%), students (21 cases, 2.1%), and children (4 cases, 0.4%). The age of malaria cases ranged from 9 months to 69 years, and most of the cases (82.6%) were in the 21–50 years age group (Table 1).

**Table 1:** Demographic characteristics of malaria cases in Zhejiang Province, 2012–2016

Variables	2012	2013	2014	2015	2016
Gender					
Male	123 (87.9%)	187 (89.9%)	195 (90.7%)	173 (89.2%)	223 (90.7%)
Female	17 (12.1%)	21 (10.1%)	20 (9.3%)	21 (10.8%)	23 (9.3%)
Age groups (years)					
≤ 20	12 (8.6%)	6 (2.9%)	7 (3.3%)	1 (0.5%)	2 (0.8%)
21–30	31 (22.1%)	54 (26.0%)	59 (27.4%)	45 (23.2%)	56 (22.8%)
31–40	47 (33.6%)	37 (17.8%)	56 (26.0%)	53 (27.3%)	65 (26.4%)
41–50	34 (24.3%)	74 (35.6%)	67 (31.2%)	65 (33.5%)	85 (34.6%)
≥ 51	16 (11.4%)	37 (17.8%)	26 (12.1%)	30 (15.5%)	38 (15.4%)
Occupation					
Businessmen	54 (38.6%)	59 (28.4%)	69 (32.1%)	65 (33.5%)	84 (34.1%)
Workers	30 (21.4%)	48 (23.1%)	41 (19.1%)	36 (18.6%)	38 (15.4%)
Farmers	24 (17.1%)	46 (22.1%)	35 (16.3%)	46 (23.7%)	56 (22.8%)
Overseas laborers	13 (9.3%)	16 (7.7%)	42 (19.5%)	14 (7.2%)	32 (13.0%)
Houseworkers	6 (4.3%)	6 (2.9%)	5 (2.3%)	5 (2.6%)	12 (4.9%)
Students	6 (4.3%)	3 (1.4%)	3 (1.4%)	5 (2.6%)	4 (1.6%)
Children	1 (0.7%)	2 (1.0%)	0	1 (0.5%)	0
Others*	6 (4.3%)	28 (13.5%)	20 (9.3%)	22 (11.3%)	20 (8.1%)

* Others include fishermen, teachers, drivers, translators, and so on.

As shown in Table 2, between 2012 and 2016, the largest number of imported cases originated in Africa (898/1,003, 89.5%), followed by Asia (100/1,003, 10.0%) and Oceania (5/1,003, 0.5%), including six cases from other provinces of China. Imported malaria cases from Africa were mainly infected in Nigeria (197/898, 21.9%), Ghana (99/898, 11.0%), Angola (98/898, 10.9%), and Equatorial Guinea (96/898, 10.7%). Cases imported from Asian countries were mainly from Myanmar (24/100, 24.0%), Pakistan (20/100, 20.0%), and India (17/100, 17.0%). By *Plasmodium* species type, 99.0% *P. falciparum* cases (757/765), 98.5% *P. ovale* cases (65/66), and 100% *P. malariae* cases (14/14) were from Africa, and *P. vivax* cases from Asia accounted for 62.3% (91/146) of all *P. vivax* cases.

**Table 2:** Origins of malaria cases in Zhejiang Province from 2012 to 2016, by *Plasmodium* species and countries

Country		*Plasmodium* species					Total
		*Plasmodium falciparum*	*Plasmodium vivax*	*Plasmodium ovale*	*Plasmodium malariae*	Mixed infection	
Africa	Nigeria	173	6	13	4	1	197
	Ghana	85	4	9	0	1	99
	Angola	85	2	7	3	1	98
	Equatorial Guinea	78	7	9	0	2	96
	Congo	62	4	7	2	1	76
	Cameron	58	0	5	1	1	65
	Liberia	14	4	3	1	2	24
	Guinea	18	1	1	0	0	20
	Cote d’Ivoire	18	0	2	0	0	20
	Mozambique	19	0	0	0	0	19
	Gabon	16	1	0	0	0	17
	Ethiopia	3	11	0	1	1	16
	Tanzania	16	0	0	0	0	16
	Uganda	10	1	2	0	1	14
	Malawi	11	2	0	0	0	13
	Sudan	8	3	0	0	0	11
	Benin	8	1	2	0	0	11
	Other African countries	75	4	5	2	0	86
Asia	Myanmar	3	20	0	0	1	24
	Pakistan	0	20	0	0	0	20
	India	0	17	0	0	0	17
	Indonesia	0	12	0	0	0	12
	Cambodia	1	10	0	0	0	11
	Vietnam	2	2	0	0	0	4
	Other Asian countries	1	4	1	0	0	6
	Other provinces of China	0	6	0	0	0	6
Oceania	Papua New Guinea	1	2	0	0	0	3
	Solomon Islands	0	2	0	0	0	2
Total		765	146	66	14	12	1,003

Based on 1,003 malaria cases, the median time from illness onset to confirmation was 3 days (range: 0–56 days). The interval time from illness onset to confirmation between cases with and without complications, cases from different imported areas, and among different *Plasmodium* species was found to be statistically significant (*P* < 0.05, summarized in Table 3). A multiple linear model was used to study the associations of time from illness onset to confirmation with age, gender, educational level, illness history, complications, *Plasmodium* species, and imported areas. The time interval was significantly associated with the complications of patients (*P* < 0.05, summarized in Table 4). The time interval from illness onset to confirmation in imported cases with and without complications was 4 days (range: 0–37 days) and 3 days (range: 0–56 days), respectively. However, no significant association was found between the time interval and age, gender, educational level, illness history, *Plasmodium* species, and imported areas (*P* > 0.05).

**Table 3:** Time from illness onset to confirmation of malaria cases in Zhejiang Province, 2012–2016

Variables	Median (days)	Range	*P* value
Overall	3	0–56	
Age (years)			0.251
< 30	3	0–48	
30–40	3	0–56	
> 40	3	0–41	
Gender			0.441
Male	3	0–56	
Female	3	0–26	
Educational level			0.196
Primary school or below	3	0–41	
Middle and high schools	2.5	0–56	
College or above	3	0–48	
Illness history			0.307
No	3	0–41	
Yes	3	0–56	
Complications			0.002
No	3	0–56	
Yes	4	0–37	
*Plasmodium* species			0.004
*Plasmodium falciparum*	3	0–48	
*Plasmodium vivax*	4	0–56	
*Plasmodium ovale*	3	0–49	
*Plasmodium malariae*	6	0–16	
Mixed infection	4	0–26	
Imported areas			0.006
Africa	3	0–49	
Asia	4	0–56	
Oceania	5	3–6	

**Table 4:** Factors affecting time interval from illness onset to confirmation of malaria cases in Zhejiang Province, 2012–2016

Independent variables*	Time interval from illness onset to confirmation	
	95% confidence interval†	*P* value
Age	0.009 (−0.027, 0.046)	0.616
Gender	−0.747 (−2.010, 0.515)	0.246
Educational level	−0.137 (−0.811, 0.536)	0.689
Illness history	−0.071 (−0.838, 0.695)	0.855
Complications	1.712 (0.325, 3.100)	0.016
*Plasmodium* species	0.496 (−0.026, 1.018)	0.062
Imported areas	0.824 (−0.369, 2.017)	0.176

* Dependent variables were defined as gender: 0=male, 1= female; educational level: 0=primary school or below, 1=middle and high schools, 2=college or above; illness history: 0=no, 1=yes; complications: 0=no, 1=yes; *Plasmodium* species: 1=*P. falciparum*, 2=*P. vivax*, 3=*P. ovale*, 4=*P. malariae, 5=*mixed infection; imported area: 0=Africa, 1=Asia, 2=Oceania.

† The linear regression coefficient.

To prevent onward transmission, RACD screening was carried out for people in contact with the malaria cases, such as coworkers who traveled to the same area (inactive foci), family members, neighbors, and others (active foci). Of the 41,560 individuals in geographic or demographic contact with the cases from 2013 to 2016, 8.3% (3,461/41,560), consisting of those with fever, were tested for malaria infection (Table 5). A total of 10 malaria-positive individuals were identified during RACD events from 2013 to 2016, including two *P. vivax* cases and eight *P. falciparum* cases. No secondary cases had occurred.

**Table 5:** Investigation of foci in Zhejiang Province, 2013–2016

	Contacts of cases	Febrile population tested	Positive individuals
2013	14,033	1,355	4
2014	11,173	1,116	2
2015	9,461	597	1
2016	6,893	393	3
Total	41,560	3,461	10

## DISCUSSION

Historically, *P. vivax* has been the predominant *Plasmodium* species in Zhejiang Province,^4^ but now *P. falciparum* is the predominant species, and *P. vivax* cases declined dramatically. This situation is similar to the *Plasmodium* species distribution in China,^6^ where the local malaria situation has been effectively controlled and indigenous cases decreased sharply.

The annual number of cases imported from other provinces reflected the decrease in indigenous cases in other areas of China. After the control and management of the infection source, Zhejiang Province coordinated with other parts of China in investigating the foci, health education, and screening of malaria among patients with fever. For imported cases from abroad, many departments collaborated with each other to prevent and control malaria among immigrants. For example, departments of entry–exit inspection were in charge of screening of malaria among patients with fever, and the information was reported in a timely manner to medical departments.

The proportion of both *P. ovale* and *P. malariae* cases increased during the study period, which were easily misdiagnosed as *P. vivax* and *P. falciparum* by morphology.^7^ Misdiagnosis can lead to the misuse of antimalarial drugs and increase the occurrence of severe cases and even deaths. Ensuring high-capacity potential for the diagnosis will facilitate early detection and standard treatment effectively for malaria cases. Moreover, in some *P. vivax* and *P. ovale* cases, it is seen that not taking medicines during the radical treatment that protect against the relapse is a problem. Therefore, clinicians and public health workers should strengthen the follow-up treatment of *P. vivax* and *P. ovale*.

The result of our study also showed that there were more male cases than female ones and that businessmen, workers, farmers, and overseas laborers were at high risk of malaria infection from 2012 to 2016. The characteristics of malaria cases are mainly because of the economic globalization and increase in population traveling to malaria-endemic areas for labor, trade, tourism, and other purposes. For the same reason, malaria was most commonly reported in adults aged between 21 and 50 years. These people were engaged in outdoor activities, which increased the risk of mosquito bites. Previous studies revealed that exported laborers generally had poor level of knowledge and awareness of malaria transmission and prevention.^8–10^ Given the fact that increased number of imported malaria into the nonendemic regions has become a great challenge to the public health,^11–13^ the awareness of malaria risk needs to be strengthened toward laborers and travelers through health education and prevention activities.

*Plasmodium falciparum* cases were mostly imported from Africa, and most *P. vivax* cases came from Asia, including six cases from other provinces of China. Globally, *P. falciparum* is prevalent in sub-Saharan Africa, whereas *P. vivax* is endemic in many parts of Asia, Oceania, and Central and South America^1,14^ *P. ovale* and *P. malariae* cases in Zhejiang Province were all from Africa, which were mostly reported in west and sub-Saharan Africa.^15–17^ As described earlier, imported malaria has showed an increasing trend. Although there have been no secondary transmission or reintroduction of imported malaria cases, Zhejiang Province remains at risk because of the presence of mosquito vector and conducive environmental conditions.^4,18^ Furthermore, *P. falciparum* and *P. vivax* outbreaks have been reported in nonendemic areas,^19–21^ and the findings from these investigations underscore the importance of effective surveillance systems for malaria. Therefore, the surveillance activities should be strengthened and improved to ensure timely detection and prompt response to individual cases efficiently,^22,23^ especially among the traveling population after returning from malaria-endemic areas.^24–26^

Early confirmation is of vital importance for malaria control and prevention, and experts believe that delays of diagnosis and treatment are the primary cause of severe infections in nonendemic regions.^27^ In Hubei Province, interval from illness onset to confirmation was shorter in highly educated people and in severe cases, respectively.^28^ In this study, the median time from illness onset to confirmation of malaria cases was 3 days. Further analysis showed that complications may delay the diagnosis of malaria, which underscores the importance of taking a detailed travel history when evaluating unexplained fever and considering malaria in the differential diagnosis. Although most malaria cases were appropriately treated, better knowledge of clinicians about malaria symptoms may reduce delay in access to effective therapy and avoid development of severe disease.

Comprehensive interventions have been conducted to prevent malaria epidemics in China. The 1–3–7 approach was a RACD program to deliver and monitor targeted interventions to those at risk when cases were identified: reporting of malaria cases within 1 day, case confirmation and investigation within 3 days, and appropriate foci response to prevent further transmission within 7 days.^23,29^ During investigation of foci, contacts of detected cases were screened for infection using microscopy and RDTs. Meanwhile, filter paper blood spots were collected for PCR to detect low-density infections that might be missed. Reactive case detection screening in our study detected 10 positive cases. Cheng et al.^30^ revealed that sensitive molecular technology such as PCR testing provides a more accurate diagnosis, and more techniques are also required for highly sensitive and effective diagnostic approaches for RACD activities.^31^

There are several limitations in our study. First, all data were collected from the ISPDCP database. The system used electronic management of data from prevention agencies, allowing real-time understanding of the local malaria situation. However, some missing information was identified in ISPDCP because it was only put into use in 2012 across the country. Furthermore, the investigation information of malaria cases collected in the surveillance system has been improved over this period; hence, some malaria case data were subsequently added to the database. Second, ISPDCP is a passive surveillance system. Some factors including detection capability, reporting methods, and availability of health facilities may influence the data quality.

Malaria importation has become a great challenge to malaria elimination in Zhejiang Province. Effective management of surveillance and response system is important for the identification of malaria cases, and medical technical training in malaria diagnosis and response still requires improvement. Departments of travel, commerce, entry–exit inspection, and health should cooperate to strengthen malaria monitoring and screening of laborers and travelers from malaria-endemic areas for the goal of prevention of local reintroduction caused by imported cases.
